# Predictors of objective cognitive impairment and subjective cognitive complaints in patients with Fabry disease

**DOI:** 10.1038/s41598-018-37320-0

**Published:** 2019-01-17

**Authors:** Simon Körver, Gert J. Geurtsen, Carla E. M. Hollak, Ivo N. van Schaik, Maria G. F. Longo, Marjana R. Lima, Leonardo Vedolin, Marcel G. W. Dijkgraaf, Mirjam Langeveld

**Affiliations:** 1 Department of Endocrinology and Metabolism, Amsterdam University Medical Centers location Academic Medical Center, Amsterdam, The Netherlands; 2 Department of Medical Psychology, Amsterdam University Medical Centers location Academic Medical Center, Amsterdam, The Netherlands; 3 Department of Neurology, Amsterdam University Medical Centers location Academic Medical Center, Amsterdam, The Netherlands; 4 Department of Radiology, Massachusetts General Hospital, Boston, United States; 5 Department of Radiology, Hospital Moinhos de Vento, Porto Alegre, Brazil; 6 Imaging Section, DASA, São Paulo Brazil; 7 Clinical Research Unit/Department of Clinical Epidemiology, Biostatistics and Bioinformatics, Amsterdam University Medical Centers location Academic Medical Center, Amsterdam, The Netherlands

## Abstract

This study investigates the relationship between objective cognitive impairment (OCI), subjective cognitive complaints and depressive symptoms in men and women with classical and non-classical Fabry disease (FD). Cognitive functioning was assessed using a neuropsychological test battery, subjective cognitive complaints using a structured interview and depressive symptoms using a depression scale (CESD). Eighty-one patients were included (mean age 44.5 ± 14.3, 35% men, 74% classical). Subjective cognitive complaints were reported by 64% of all patients. OCI was present in thirteen patients (16%), predominantly in men with classical FD. Thirty-one patients (38%) had a high score (≥16) on the CESD scale. Male sex (OR, 6.8; 95%CI, 1.6–39.8; p = 1.6 * 10^−2^) and stroke (OR, 6.4; 95% CI, 1.1–41.0; p = 3.7 * 10^−2^) were independently positively associated with OCI, and premorbid IQ (one IQ point increase: OR, 0.91; 95%CI, 0.82–0.98; p = 3.8 * 10^−2^) was independently negatively associated with OCI. The CESD-score (one point increase: OR, 1.07; 95% CI, 1.02–1.13; p = 3.3 * 10^−3^) and a history of depression (OR, 2.7; 95% CI, 1.1–7.3; p = 3.9 * 10^−2^) were independently positively associated with subjective cognitive complaints. OCI is present in 16% of FD patients, warranting referral for neuropsychological assessment. Nevertheless, subjective cognitive complaints are related to depressive symptoms, emphasizing the importance of recognition and treatment of the latter.

## Introduction

Fabry disease (FD; OMIM 301500) is a rare, X-linked, lysosomal storage disorder. A mutation in the GLA-gene causes a lack or absence of enzymatic activity of α-galactosidase A resulting in the accumulation of glycosphingolipids, mainly affecting the cardiovascular and nervous system. Sex and phenotype have been determined as important predictors of the disease course in FD^1^. Women generally have a more attenuated disease course compared to men^1^. Phenotypically, FD can be split in a milder, non-classical and a classical, more severe phenotype, with multi-organ involvement^2^.

Frequent cerebral manifestations of FD are the occurrence of white matter lesions (WMLs), TIA and stroke. Furthermore, depressive symptoms are highly prevalent^3^. WMLs, stroke and depression in itself are known to result in objective cognitive impairment (OCI) in the general population^4^. Previous studies on OCI in FD were limited by small sample size, often did not incorporate neuroimaging, combined WMLs and stroke as a single entity and did not differentiate between patient groups with different FD phenotypes and sex^3,5–7^. Small study populations also restricted the identification of variables related to OCI in FD.

Subjective cognitive complaints are frequently mentioned by FD patients during their routine clinic visits and were related to depressive symptoms but not to OCI in a recent study in a FD population^7^. Extending knowledge on the relationship between subjective cognitive complaints and depressive symptoms and/or OCI can have significant implications for the therapeutic measures indicated to address these complaints. The objective of this study was to investigate the prevalence of OCI, subjective cognitive complaints, depressive symptoms and to explore their risk factors and interrelation in patients with FD in general as well as in subgroups defined by sex and FD phenotype.

## Results

### Baseline characteristics

Of the 154 known FD patients in the AMC, ten patients were not considered eligible because of comorbidity known to influence the neuropsychological test results (autism (n = 2), blindness (n = 1), intellectual and developmental disabilities (n = 3), severe aphasia (n = 1)) or because of insufficient knowledge of the Dutch language (n = 3).

Of the 144 contacted patients, 63 patients were not willing to participate (not interested (n = 29), time constraints (n = 8) and participation being too strenuous (n = 26)). There were no significant differences between participants and non-participants in sex, phenotype, age, TIA, stroke or median Fazekas score (see Supplemental Table E-4 for data on non-participants). A total of 81 patients were included with a mean age of 44.5 ± 14.3 years (range: 19–76 years) (Table 1). Fifty-three patients were women (65%) and 60 patients had classical FD phenotype (74%). Twenty-two patients (27%) reported a history of or a current depression, as diagnosed by their general practitioner, psychologist or psychiatrist, without statistically significant differences between subgroups split by sex and phenotype (p = 6.6 * 10^−1^).

**Table 1:** Patient characteristics.

	All	Men		Women		Intergroup comparison	
		Classical (a)	Non-classical (b)	Classical (c)	Non-classical (d)	P	Post hoc
Patients, n (%)	81	17 (21.0%)	11 (13.6%)	43 (53.1%)	10 (12.3%)	—	—
Age in years, mean (±SD)	44.5 (±14.3)	38.6 (±13.5)	58.0 (±11.2)	43.5 (±13.9)	43.9 (±13.0)	**3.5** * **10**^**−3**^	a,c < b
History of ERT, n (%)	48 (59.3%)	17 (100.0%)	3 (27.3%)	27 (62.8%)	1 (10.0%)	**5.3** * **10**^**−7**^	b,c,d < a, d < c
Currently on ERT, n (%)	43 (53.1%)	15 (88.2%)	2 (18.2%)	25 (58.1%)	1 (10.0%)	**3.2** * **10**^**−5**^	d < a,c, b < a
Replagal/Fabrazyme, n/n	11/32	5/10	0/2	6/19	0/1	—	—
Time on ERT in years, median (range)	8.6 (0.1–16.0)	12.4 (1.6–16.0)	9.5 (6.4–12.5)	7.6 (0.1–13.6)	0.2	6.4 * 10^**−**2^	—
Current psychiatric medication, n (%)	15 (18.5%)	2 (11.8%)	3 (27.3%)	9 (20.9%)	1 (10.0%)	6.5 * 10^**−**1^	—
Antidepressants^†^, n (%)	7 (8.6%)	1 (5.9%)	2 (18.2%)	3 (7.0%)	1 (10.0%)	6.4 * 10^**−**1^	—
Benzodiazepines, n (%)	9 (11.1%)	1 (5.9%)	1 (9.1%)	7 (16.3%)	0 (0.0%)	5.9 * 10^**−**1^	—
Unemployed^%^, n (%)	32 (39.5%)	9 (52.9%)	5 (45.5%)	15 (34.9%)	3 (30.0%)	5.4 * 10^**−**1^	—
Unfit for work^$^, n (%)	20 (24.7%)	7 (41.2%)	2 (18.1%)	10 (23.3%)	1 (10.0%)	3.1 * 10^**−**1^	—
Single^#^, n (%)	30 (37.0%)	9 (52.9%)	4 (36.4%)	14 (32.6%)	3 (30.0%)	5.1 * 10^**−**1^	—
Years of education, mean (±SD)	13.8 ± 3.0	14.4 ± 2.8	13.9 ± 4.9	13.3 ± 2.7	14.9 ± 1.8	3.5 * 10^**−**1^	—
Depression*, n (%)	22 (27.2%)	3 (17.6%)	3 (27.3%)	12 (27.9%)	4 (40.0%)	6.6 * 10^**−**1^	—
Burnout*, n (%)	12 (14.8%)	1 (5.9%)	0 (0.0%)	7 (16.3%)	4 (40.0%)	5.8 * 10^**−**2^	—
Smoking, n (%)	36 (44.4%)	6 (35.3%)	6 (54.5%)	21 (48.8%)	3 (30.0%)	5.6 * 10^**−**1^	—
Hypertension, n (%)	24 (29.6%)	2 (11.8%)	7 (63.6%)	14 (32.6%)	1 (10.0%)	**1.5** * **10**^**−2**^	‡
Diabetes mellitus type 2, n (%)	3 (3.7%)	0 (0.0%)	1 (9.1%)	2 (4.7%)	0 (0.0%)	1.0 * 10^0^	—
Dyslipidemia, n (%)	11 (13.6%)	1 (5.9%)	4 (36.4%)	6 (14.0%)	0 (0.0%)	8.1 * 10^**−**2^	—
eGFR in ml/min/1.73 m^2^, median (range)	94.6 (11.4–141.0)	105.6 (25.4–141.0)	77.3 (11.4–109.9)	94.0 (45.6–131.1)	95.4 (73.6–118.3)	**4.1** * **10**^**−3**^	b < a,c,d
eGFR <60 ml/min/1.73 m^2^, n (%)	11 (13.6%)	2 (11.8%)	4 (36.4%)	5 (11.6%)	0 (0.0%)	1.2*10^**−**1^	—
Albuminuria in mg/day, median (range)	32 (3–2761)	60 (5–921)	100 (7–2761)	21 (3–1426)	13.5 (4–1422)	7.4 * 10^**−**2^	—
Albuminuria >A1	41 (50.6%)	12 (70.6%)	7 (63.6%)	21 (48.8%)	1 (10.0%)	**1.5** * **10**^**−2**^	d < a
LVMI in gr/m^2^, median (range)	62.7 (33.4–139.6)	78.3 (45.9–139.5)	64.7 (50.1–136.9)	55.9 (36.6–119.1)	44.7 (33.4–77.6)	**1.5** * **10**^**−4**^	c < a, d < a,b
LVMI >upper ref limit, n (%)	24/74 (32.4%)	9/17 (52.9%)	3/8 (37.5%)	11/39 (28.2%)	1/10 (10.0%)	1.2 * 10^**−**1^	—
Cardiac fibrosis	23/72 (31.9%)	6/17 (35.3%)	2/6 (33.3%)	14/39 (35.9%)	1/10 (10.0%)	4.4 * 10^**−**1^	—
LysoGb3 before ERT in nmol/L, median (range)	8.2 (0.6–150.3)	99.0 (36.8–150.3)	5.0 (1.2–16.5)	7.8 (1.3–22.6)	1.9 (0.6–5.0)	**5.5** * **10**^**−11**^	b,c,d < a, d < c

Continuous variables are presented as median (range) or mean (±SD) and discrete variables as number (percentages). Intergroup differences were tested, results <0.05 are in bold. If <0.05 then post-hoc tests were performed. For representation of the results of the post-hoc analyses we allocated a letter (a,b,c or d) to each subgroup.

ERT = enzyme replacement therapy, eGFR = estimated glomerular filtration rate, LVMI = left ventricular mass index.

^†^Antidepressants taken for neuropathic pain not included, ^%^Includes three retirees, ^$^Includes three patients regarded partially unfit for work, ^#^Divorced, widowed, or no romantic relationship.

*History of or current, as diagnosed by a general practitioner, psychologist or psychiatrist, ^‡^Post-hoc Fisher’s exact test was not significant after Bonferroni-Holm correction.

Upper reference limit LVM: ♂ = 79/♀ = 75. Normal range lysoGb3 = 0.3–0.6 nmol/L. Albuminuria >A1 = > 30 mg/day.

### Subjective cognitive complaints

Fifty-two patients (64%) experienced subjective cognitive complaints in at least one domain, without statistically significant differences between subgroups split by sex and phenotype (p = 3.0 * 10^−1^) (Table 2).

**Table 2:** Number of patients with subjective cognitive complaints, objective cognitive impairment and T-scores ≤33.

	All	Men		Women	
		Classical	Non-classical	Classical	Non-classical
**Cognitive dysfunction**					
Subjective cognitive complaints^&^	52 (64.2%)	11 (64.7%)	5 (45.5%)	31 (71.1%)	5 (50.0%)
Any OCI	13 (16.0%)	7 (41.0%)	3 (27.3%)	3 (7.0%)	0 (0%)
Severe OCI	4 (4.9%)	2 (11.8%)	1 (9.1%)	1 (2.3%)	0 (0%)
	T≤33, n (%)	T≤33, n (%)	T≤33, n (%)	T≤33, n (%)	T≤33, n (%)
**Intelligence estimation**					
DART*	3 (3.8%)	0 (0%)	3 (27.3%)	0 (0%)	0 (0%)
**Language**					
BNT	0 (0%)	0 (0%)	0 (0%)	0 (0%)	0 (0%)
WAIS-IV: S*	5 (6.3%)	0 (0%)	1 (9.1%)	4 (9.5%)	0 (0%)
**Memory**					
RAVLT ir	6 (7.4%)	4 (23.5%)	1 (9.1%)	1 (2.3%)	0 (0%)
RAVLT dr	4 (4.9%)	3 (17.6%)	0 (0%)	1 (2.3%)	0 (0%)
RBMT ir	2 (2.5%)	2 (11.7%)	0 (0%)	0 (0%)	0 (0%)
RBMT dr	3 (3.7%)	2 (11.7%)	0 (0%)	1 (2.3%)	0 (0%)
**Visuospatial perception**					
WAIS-IV: BD*	6 (7.5%)	2 (11.7%)	0 (0%)	3 (7.1%)	1 (10%)
JLO	3 (3.7%)	0 (0%)	1 (9.1%)	2 (4.7%)	0 (0%)
**Processing speed**					
TMT A	0 (0%)	0 (0%)	0 (0%)	0 (0%)	0 (0%)
Stroop W	0 (0%)	0 (0%)	0 (0%)	0 (0%)	0 (0%)
Stroop C	4 (4.9%)	2 (11.7%)	1 (9.1%)	1 (2.3%)	0 (0%)
**Attention and executive functioning**					
TMT B	3 (3.7%)	1 (5.9%)	0 (0%)	2 (4.7%)	0 (0%)
Stroop CW	2 (2.5%)	0 (0%)	1 (9.1%)	1 (2.3%)	0 (0%)
Fluency A	10 (12.3%)	3 (17.6%)	1 (9.1%)	5 (11.6%)	1 (10%)
Fluency O	11 (13.6%)	6 (35.3%)	0 (0%)	5 (11.6%)	0 (0%)
Fluency L	7 (8.6%)	2 (11.7%)	4 (36.3%)	1 (2.3%)	0 (0%)

Discrete variables as number (percentages).

OCI = objective cognitive impairment, DART = Dutch Adult Reading Test, BNT = Boston Naming Test, WAIS-IV: S = Wechsler Adult Intelligence Scale IV: Similarities, RAVLT = Rey Auditory Verbal Learning Test, ir = immediate recall, dr = delayed recall, RBMT = Rivermead Behavioural Memory Test, BD = Block Design, JLO = Judgement of Line Orientation, TMT = Trail Making Test, W = Words, C = Color, CW = Color-Word, A = Animal, O = Occupation, L = Letter. & Presence of subjective cognitive complaints on memory, attention and/or executive functioning in at least one domain,  *One woman with classical Fabry disease used the DART, WAIS-IV: S and WAIS-IV: BD in her job setting so did not perform these cognitive tests.

### Objective cognitive impairment

There were no signs of underachievement or lack of motivation in any of the patients based on the TOMM score.

A total of 13 (16%) patients had any OCI, of which four (5%) had severe OCI (Table 2). Seven men with classical FD (41%) had any OCI of which two (12%) had severe OCI. For men with non-classical FD this was three (27%) and one (9%), respectively, and in women with classical FD this was three (7%) and one (2%), respectively. OCI did not occur in women with non-classical FD.

Most abnormal T-scores (T-scores ≤33) were found in the attention and executive functioning domain (Table 2). Decreased T-scores were found in the attention and executive functioning domain in men with classical FD (T-score, 45.6; p = 1.4 * 10^−2^) and men with non-classical FD (T-score, 46.6; p = 1.2 * 10^−2^) (Table 3). None of the patients scored <24 on the MMSE, suggesting lack of sensitivity for the detection of OCI in FD using this cut-off. After post-hoc correction there were no differences in premorbid IQ between the subgroups divided by sex and phenotype (Table 3). However, there was a difference looking at sex only: men had a lower premorbid IQ compared to women (W = 468.5, p = 6.6 * 10^−3^).

**Table 3:** Results cognitive subtests/domains and subgroup comparison.

	All	Men		Women		Intergroup comparison	
		Classical (a)	Non-classical (b)	Classical (c)	Non-classical (d)	P	Post-hoc
**General cognitve functioning**							
MMSE^†^	29 (25–30)	29 (27–30)	29 (27–30)	29 (25–30)	29 (28–30)	—	—
**Intelligence estimation**							
DART^#,^*	94.0 (68–133)	89.0 (83–114)	85.0 (68–133)	94.5 (82–121)	100.0 (84–121)	**4.4** * **10**^**−2**^	‡
Language*	49.5 (32.0–63.0)	51.5 (39.5–62.0)	45.5 (36.0–61.5)	48.8 (32.0–59.5)	55.0 (42.0–63.0)	7.1 * 10^**−**2^	—
BNT	50.0 (37–63)	54.0 (39–63)	46.0 (39–63)	50.0 (37–59)	47.5 (37–63)	4.9 * 10^**−**1^	—
WAIS-IV: S*	50.0 (27–72)	50.0 (40–72)	44.0 (33–60)	50.0 (27–63)	59.0 (40–70)	**2.3** * **10**^**−2**^	b < d
**Memory**	55.0 (22.8–71.5)	54.5 (22.8–69.5)	55.3 (38.5–64.3)	54.8 (24.8–71.5)	57.8 (42.8–71.0)	2.2 * 10^**−**1^	—
RAVLT ir	52.0 (16–72)	49.0 (18–65)	57.0 (32–66)	52.0 (16–68)	57.5 (47–72)	3.9 * 10^**−**2^	—
RAVLT dr	53.0 (21–71)	48.0 (21–69)	54.0 (34–64)	53.0 (27–71)	56.0 (44–64)	3.7 * 10^**−**1^	—
RBMT ir	57.0 (27–81)	59.0 (27–73)	57.0 (34–68)	57.0 (34–81)	58.0 (41–75)	3.2 * 10^**−**1^	—
RBMT dr	55.0 (22–76)	54.0 (25–76)	59.0 (41–69)	54.0 (22–74)	56.5 (39–75)	8.0 * 10^**−**1^	—
Visuospatial perception*	54.0 (28.0–65.5)	54.5 (44.5–64.0)	48.0 (36.5–54.0)	55.8 (28.0–65.5)	58.5 (47.0–65.5)	**6.1** * **10**^**−3**^	b < a,c,d
WAIS-IV BD*	50.0 (27–72)	50.0 (33–67)	*43.0 (34–50)* ^a^	52.0 (27–72)	60.0 (33–70)	2.8 * 10^**−**2^	—
JLO	61.0 (29–61)	61.0 (52–61)	52.0 (33–61)	61.0 (29–61)	61.0 (48–61)	7.9 * 10^**−**2^	—
**Processing speed**	53.7 (32.3–74.7)	49.7 (42.0–60.0)	55.7 (40.3–74.7)	52.7 (32.3–63.3)	54.3 (45.7–70.3)	3.4 * 10^**−**2^	—
TMT A	56.0 (34–77)	51.0 (38–61)	55.0 (34–63)	56.0 (34–77)	59.5 (43–71)	9.0 * 10^**−**2^	—
Stroop W	56.0 (34–84)	56.0 (41–69)	51.0 (41–61)	60.0 (37–84)	54.0 (41–77)	1.9 * 10^**−**2^	—
Stroop C	52.0 (29–88)	47.0 (29–59)	53.0 (29–88)	53.0 (33–71)	52.0 (39–71)	1.0 * 10^**−**1^	—
**Attention and executive functioning**	48.8 (25.6–66.8)	*45.6 (35*.2*–58.8)*^*b*^	*46.6 (*3*7.2–55*.4*)*^*c*^	50.2 (25.6–66.0)	52.6 (40.2–66.8)	8.3 * 10^**−**2^	—
TMT B	51.0 (−1–74)	47.0 (33–58)	49.0 (35–54)	51.0 (−1–74)	51.0 (42–59)	2.8 * 10^**−**1^	—
Stroop CW	50.0 (32–84)	48.0 (39–60)	43.0 (33–61)	51.0 (32–84)	53.5 (45–71)	1.5 * 10^**−**2^	—
Fluency A	50.0 (29–75)	48.0 (29–75)	54.0 (29–61)	50.0 (29–69)	57.0 (29–69)	4.9 * 10^**−**1^	—
Fluency O	50.0 (17–69)	43.0 (24–64)	50.0 (38–57)	48.0 (17–69)	56.0 (36–67)	1.3 * 10^**−**1^	—
Fluency L	*4*5*.0 (25–71)*^*d*^	*42.0 (25–*6*4)*^*e*^	*43.0 (27–60)* ^*f*^	50.0 (33–71)	43.5 (36–68)	1.7*10 ^**−3**^	a,b < c

All variables are presented as median (range). The MMSE is presented as a raw score with range (<24 is considered a diagnostic clue for the presence of dementia), the DART is presented as IQ-score, all other results are presented as T-scores in comparison to the reference population, where the mean is 50 and one SD is 10. T-scores <50 were compared to a T-score of 50 and presented in italics if they were statistically significant after Bonferroni-Holm correction. Intergroup differences were compared. Significant results (after Bonferroni-Holm correction) are in bold and were followed by post-hoc testing. For representation of the results of the post-hoc analyses we allocated a letter (a, b, c or d) to each subgroup.

MMSE = Mini Mental State Exam, DART = Dutch Adult Reading Test, BNT = Boston Naming Test, WAIS-IV: S = Wechsler Adult Intelligence Scale IV: Similarities, RAVLT = Rey Auditory Verbal Learning Test, ir = immediate recall, dr = delayed recall, RBMT = Rivermead Behavioural Memory Test, BD = Block Design, JLO = Judgement of Line Orientation, TMT = Trail Making Test, W = Words, C = Color, CW = Color-Word, A = Animal, O = Occupation, L = Letter.

^†^Raw MMSE score, ^#^IQ score, *One classical women used the DART, WAIS-IV: S and WAIS-IV: BD in her job setting so did not perform these cognitive tests, ^‡^Post-hoc Tukeys test was not significant.

^a^p = 9.8 * 10^**−**4^, ^b^p = 1.4 * 10^**−**2^, ^c^p = 1.2 * 10^**−**2^, ^d^p = 8.4  * 10^**−**3^, ^e^p = 6.4 * 10^**−**3^, ^f^p = 5.9 * 10^**−**3^.

### Questionnaires

Thirty-one patients (38.3%) scored ≥16 on the CESD, indicating the presence of depressive symptoms (Table 4), with comparable scores in all subgroups. MSSI scores were higher in men and women with classical FD and men with non-classical disease compared to women with non-classical disease, indicating less severe disease in women with non-classical disease. BPI, MCS and PCS scores were comparable in all subgroups, indicating no differences in pain, mental QoL and physical QoL in all subgroups, respectively. Almost half of all patients (n = 39) experienced poor sleep quality.

**Table 4:** Questionnaires and indexes.

	All	Men		Women		Intergroup comparison	
		Classical (a)	Non-classical (b)	Classical (c)	Non-classical (d)	P	Post hoc
CESD, median (range)	11 (0–44)	11 (0–40)	12 (0–37)	12 (0–44)	7.5 (0–20)	6.3 * 10^**−**1^	—
CESD ≥16, n (%)	31 (38.3%)	7 (41.2%)	4 (36.4%)	17 (39.5%)	3 (30.0%)	9.7 * 10^**−**1^	
MSSI, median (range)	24 (2–68)	32 (15–68)	24 (2–41)	23 (4–42)	6.5 (2–20)	**3.3** * **10**^**−5**^	d < a,b,c, c < a
MSSI 20–40, n (%)	48 (59.3%)	13 (76.5%)	6 (54.5%)	28 (65.1%)	1 (10.0%)	—	—
MSSI >40, n (%)	7 (8.6%)	2 (11.8%)	2 (18.2%)	1 (2.3%)	0 (0.0%)	—	—
BPI worst, median (range)	2 (0–8)	2 (0–7)	4 (0–7)	3 (0–8)	0 (0–8)	8.4 * 10^**−**1^	—
BPI average, median (range)	1 (0–7)	1 (0–7)	4 (0–7)	2 (0–7)	0 (0–6)	6.6 * 10^**−**1^	—
BPI interference, median (range)	0.9 (0.0–6.9)	0.4 (0.0–5.0)	3.3 (0.6–6.4)	0.9 (0.0–6.9)	0.1 (0.0–6.3)	5.0 * 10^**−**1^	—
PCS, median (range)	43.5 (18.4–62.9)	39.8 (20.6–62.9)	39.9 (18.4–60.2)	42.4 (22.5–59.6)	52.3 (24.1–59.4)	3.9 * 10^**−**1^	—
MCS, median (range)	49.8 (13.2–65.1)	49.0 (13.2–65.1)	46.3 (23.3–60.8)	49.0 (21.4–62.2)	54.5 (34.6–61.6)	3.3 * 10^**−**1^	—
PSQI, median (range)	5.0 (0.0–20.0)	4.0 (0.0–14.0)	6.0 (1.0–13.0)	6.0 (1.0–20.0)	5.5 (2.0–10.0)	**4.7** * **10**^**−2**^	a < c
PSQI >5, n (%)	39 (48.1%)	4 (23.5%)	7 (63.6%)	23 (53.5%)	5 (50.0%)	—	—

Continuous variables are presented as median (range), discrete variables as number (percentages). Intergroup differences were compared. Significant results (after Bonferroni-Holm correction) are in bold and were followed by post-hoc testing. For representation of the results of the post-hoc analyses we allocated letter (a, b, c or d) to each subgroup. For additional information on the interpretation of questionnaires and indexes please see: methods, questionnaires. CESD = Centre for Epidemiological Studies – Depression scale, MSSI = Mainz Severity Score Index, BPI = Brief Pain Inventory, PCS = SF-36 physical component scale, MCS = SF-36 mental component scale, PSQI = Pittsburgh Sleep Quality Index.

### Cerebral involvement

Ten patients had a history of stroke as diagnosed by a neurologist, none of them were women with non-classical FD (Table 5). Seventy-three patients (90%) had an MRI brain before the neuropsychological assessment. WMLs were present in 43 patients (58.9%) and were most often mild (total Fazekas score of 1 or 2) (n = 27) (median time between MRI and assessment: 0.7 years).

**Table 5:** Cerebral involvement and MRI brain assessment.

	All	Men		Women	
		Classical	Non-classical	Classical	Non-classical
History of cerebral event^#^, n (%)	15 (18.5%)	4 (23.5%)	2 (18.2%)	9 (20.9%)	0 (0%)
History of TIA^#^, n (%)	10 (12.3%)	3 (17.6%)	2 (18.2%)	5 (11.6%)	0 (0%)
History of stroke^#^, n (%)	10 (12.3%)	2 (11.8%)	2 (18.2%)	6 (14.0%)	0 (0%)
MRI present, n (%)	73 (90.1%)	17 (100%)	7 (63.6%)	39 (90.7%)	10 (100.0%)
Time since MRI in years, median (range)	0.7 (0.0–2.9)	0.5 (0.0–2.8)	0.6 (0.0–1.5)	0.9 (0.2–2.9)	0.9 (0.1–2.6)
WMLs present, n (%)	43 (58.9%)	9 (52.9%)	5 (71.4%)	23 (59.0%)	6 (60.0%)
Fazekas					
Total score (0–6), median (range)	1 (0–6)	0 (0–6)	1 (0–3)	1 (0–6)	0.5 (0–2)
Deep WMLs					
None (0), n (%)	38 (52.1%)	9 (52.9%)	4 (57.1%)	20 (51.3%)	5 (50.0%)
Punctate (1), n (%)	22 (30.1%)	2 (11.8%)	2 (28.6%)	13 (33.3%)	5 (50.0%)
Early confluent (2), n (%)	9 (12.3%)	4 (23.5%)	1 (14.3%)	4 (10.3%)	0 (0%)
Confluent (3), n (%)	4 (5.5%)	2 (11.8%)	0 (0%)	2 (5.1%)	0 (0%)
Periventricular WMLs					
None (0), n (%)	46 (63.0%)	11 (64.7%)	2 (28.6%)	24 (61.5%)	9 (90.0%)
Caps/lines (1), n (%)	18 (24.7%)	1 (5.9%)	5 (71.4%)	11 (28.2%)	1 (10.0%)
Bands (2), n (%)	5 (6.8%)	3 (17.6%)	0 (0%)	2 (5.1%)	0 (0%)
Irregular extending into WM (3), n (%)	4 (5.5%)	2 (11.8%)	0 (0%)	2 (5.1%)	0 (0%)
(Lacunar) stroke on MRI, n (%)	13 (17.8%)	5 (29.4%)	3 (42.9%)	4 (10.3%)	1 (10.0%)
Number of (Lacunar) stroke(s) on MRI, median (range)	0 (0–8)	0 (0–5)	0 (0–8)	0 (0–3)	0 (0–1)
BAD in mm, median (range)	3.6 (2.5–5.9)	4.2 (3.1–5.6)	3.6 (3.3–4.3)	3.6 (2.5–5.9)	3.2 (2.5–3.6)
MTA, median (range)	1 (0–3)	1 (0–3)	1 (0–2)	1 (0–3)	1 (0–2)

Continuous variables are presented as median (range), discrete variables as number (percentages).

TIA = Transient Ischemic Attack, WMLs = White matter lesions, WM = White matter, BAD = Basilar artery diameter, MTA = Medial temporal lobe atrophy scale.

^#^As diagnosed by a neurologist.

### Variables affecting cognitive domains

Higher premorbid IQ was related to increased T-scores in all five domains (Table 6). Men generally scored lower on processing speed (β = −5.37; 95%CI −8.92 to −1.81; p = 3.6 * 10^−3^) compared to women. Other factors related to a lower T-score on processing speed were the pain score (one point increase BPI interference: β = −1.31; 95%CI −2.12 to −0.51; p = 1.8 * 10^−3^), being single (β = −5.19; 95%CI −8.69 to −1.68; p = 4.3 * 10^−3^), the MSSI score (one point increase: β = −0.20; 95%CI −0.33 to −0.06; p = 4.9 * 10^−3^) and BAD (one mm increase: β = −3.68; 95%CI −5.92 to −1.17; p = 4.1 * 10^−3^). Being employed was positively related to processing speed (β = 4.94; 95%CI 1.46 to 8.42; p = 5.9 * 10^−3^).

**Table 6:** Univariate relations to subjective cognitive complaints, objective cognitive impairment and cognitive domains.

	Men	Classical	Classical men	Age (years)	Education (years)	DART (premorbid IQ)	Employment	Single	Alcohol/drug abuse	CESD	BPI interference
**Cognitive dysfunction**											
SCC	−0.097	0.255*	0.017	−0.028	−0.069	−0.008	−0.293**	0.106	0.056	0.362***	0.221*
OCI	0.389***	0.028	0.353**	0.062	−0.115	−0.288**	−0.128	0.291**	0.225*	−0.108	0.131
**Cognitive domains**											
Language	0.49	—	2.99	—	0.80**	0.32***	0.71	1.18	—1.20	−0.12	−0.725
Memory	*−2.79*	—	−4.87	—	—	0.63**	2.91	−6.22**	−7.17*	*−0.06*	−1.14*
Visuospatial perception	*−2.94*	—	1.47	—	0.71*	0.25**	−0.02	−0.18	2.66	−0.10	−1.06**
Processing speed	−5.37**	—	−5.09*	—	—	0.23**	4.94**	−5.19**	2.38	−0.19*	−1.31**
Executive functioning	−4.14*	—	−3.29	—	—	0.33***	3.78*	−5.15**	2.28	−0.12	−0.93*
	**PSQI**	**PCS**	**MCS**	**LVMI**	**eGFR**	**ERT use**	**Presence of fatigue**	**History of depression**	**MSSI**	**MSSI general**	**MSSI cardiac**
**Cognitive dysfunction**											
SCC	0.297***	−0.317***	−0.288***	−0.005	0.020	0.252*	0.287**	0.258*	0.246**	0.324***	0.103
OCI	−0.055	−0.125	0.028	0.281**	−0.102	0.074	−0.022	0.191	0.256**	0.081	0.239*
**Cognitive domains**											
Language	—	0.06	—	0.02	—	—	—	−2.07	−0.02	—	−0.12
Memory	—	*0.17*	—	−0.08*	—	—	—	*−1.60*	−0.16*	—	−0.37*
Visuospatial perception	—	0.15*	—	−0.03	—	—	—	−0.93	−0.12	—	−0.29*
Processing speed	—	0.12	—	−0.08*	—	—	—	0.60	−0.20**	—	−0.25
Executive functioning	—	0.09	—	−0.01	—	—	—	−0.88	−0.11	—	−0.08
	**MSSI Renal**		**MSSI neuro**		**Fazekas** ^**#**^		**Number of Infarctions on MRI** ^**#**^		**History of stroke**	**MTA** ^**#**^	**BAD** ^**#**^
**Cognitive dysfunction**											
SCC	0.129		0.319***		0.168		0.115		0.178	0.224*	0.160
OCI	0.201		0.150		0.218*		0.137		0.245*	0.076	0.224*
Severe OCI	—		—		0.307**		0.217		0.261*	0.215	0.210*
**Cognitive domains**											
Language	−0.02		0.04		−1.01*		−0.88		−1.94	—	−0.98
Memory	*−0.13*		*−0.40*		−1.80**		−1.99*		*−2.40*	—	−4.05**
Visuospatial perception	−0.44*		−0.07		−1.38*		−1.26		−3.87	—	−1.34
Processing speed	−0.34		−0.56**		−1.16*		−1.71*		−4.31	—	−3.68**
Executive functioning	−0.13		−0.45*		−1.06		−1.49*		−2.94	—	−1.97

Univariate relations to subjective cognitive complaints (SCC) and objective cognitive impairment (OCI) were tested using kendalls tau-b. Univariate relations to the combined T-scores of the cognitive domains were tested using generalized linear models and are presented as beta’s. For binary variables kendalls tau-b and beta’s were calculated with the presented value coded as 1 (men, classical, classical men, employment, single, alcohol/drug abuse, ERT use, presence of fatigue, history of depression and history of stroke) and the other value coded as 0 (e.g. women, non-classical, unemployment). For continuous variables beta’s were calculated for a 1 year increase (age, education), 1 point increase on IQ, index, questionnaire or scale (DART, CESD, BPI interference, PSQI, PCS, MCS, MSSI, Fazekas, MTA), 1 gr/m^2^ increase (LVMI), 1 ml/min/1.73 m^2^ increase (eGFR), 1 extra infarction (number of infarctions) and 1 mm increase (BAD).

*<0.05, **<0.01, ***<0.001. Bold printed numbers are significant after Benjamini-Hochberg correction. In several cases the T-scores of cognitive domains Memory and Visuospatial perception violated the assumptions of the generalized linear model. In these cases the T-scores were squared to satisfy these assumptions. Consequently, instead of beta’s, the difference between the square root of the predicted values is presented in italics.

MRI brain variables were also related to severe OCI.

SCC = Subjective cognitive complaints, OCI = Objective cognitive impairment, DART = Dutch adult reading test, CESD = Centre for Epidemiological Studies – Depression scale, BPI = Brief Pain Inventory, PSQI = Pittsburgh Sleep Quality Index, PCS = SF-36 physical component scale, MCS = SF-36 mental component scale, LVMI = Left ventricular mass index, eGFR = Estimated glomerular filtration rate, ERT = Enzyme replacement therapy, MSSI = Mainz Severity Score Index, MTA = Medial temporal lobe atrophy scale, BAD = Basilar artery diameter.

^#^After removal of two classical men with a history of severe drug abuse.

Being single was also negatively related to executive functioning (β = −5.15; 95%CI −8.33 to −1.97; p = 1.8 * 10^−3^).

BAD and Fazekas score were related to a lower T-score on the memory domain (BAD: increase one mm β = −4.05; 95%CI −6.64 to −0.85; p = 1.2 * 10^−2^, Fazekas: one point increase β = −1.80; 95%CI −3.10 to −0.51; p = 7.1 * 10^−3^) as was being single (β = −5.15; 95%CI −10.32 to −2.12; p = 3.4 * 10^−3^).

### Variables affecting objective cognitive impairment

Male sex was positively related to the presence of OCI (r_τ_ = 0.39; 95%CI, 0.15 to 0.58; p = 5.1 * 10^–4^) (Table 6). This relation was still present when comparing men with classical FD to all other patients (r_τ_ = 0.35; 95%CI, 0.09 to 0.60; p = 1.7 * 10^−3^). Higher premorbid IQ was negatively related to the presence of OCI (r_τ_ = −0.29; 95%CI, −0.45 to −0.079; p = 2.2 * 10^−3^). There was a positive relation between brain parameters and the presence of OCI (Fazekas score, r_τ_ = 0.22; 95%CI, −0.02 to 0.41; p = 4.7 * 10^−2^, BAD, r_τ_ = 0.22; 95%CI, −0.04 to 0.37; p = 2.1 * 10^−2^). This relation was more robust when only the relationship of severe OCI to the Fazekas score was considered (r_τ_ = 0.31; 95%CI, 0.18 to 0.41; p = 5.2 * 10^−3^). There was a positive relation between a history of stroke and the presence of OCI (r_τ_ = 0.25; 95%CI, −0.04 to 0.52; p = 2.9 * 10^−2^). There was no relation between OCI and the CESD score or between OCI and subjective cognitive complaints.

Two logistic regression models were comparable in AIC and both were in agreement with our theoretical concepts. The first model showed that male sex (OR, 6.8; 95%CI, 1.6 to 39.8; p = 1.6 * 10^−2^) and a history of stroke (OR, 6.4; 95%CI, 1.1 to 41.0; p = 3.7 * 10^−2^) were independently positively associated with OCI and that premorbid IQ (one IQ point increase: OR, 0.91; 95%CI, 0.82 to 0.98; p = 3.8 * 10^−2^) was independently negatively related to OCI. The second model showed that male sex (OR, 5.9; 95%CI, 1.4 to 31.7; p = 2.3 * 10^−2^) and being single (OR, 4.8; 95%CI, 1.2 to 25.2; p = 3.9 * 10^−2^) were both independently positively associated with OCI and that premorbid IQ (one IQ point increase: OR, 0.91; 95%CI, 0.81 to 0.99; p = 4.6 * 10^−2^) was independently negatively associated with OCI. Including male sex, a history of stroke, being single and premorbid IQ in one model did not improve the model.

### Variables affecting subjective cognitive complaints

The CESD score was most strongly positively related to subjective cognitive complaints (r_τ_ = 0.36; 95%CI, 0.18 to 0.51; p = 2.7 * 10^−5^) (Table 6). The PSQI score (r_τ_ = 0.30; 95%CI, 0.13 to 0.45; p = 7.8 * 10^−4^) and the MSSI score also showed a positive relation to subjective cognitive complaints, the latter relation was mostly driven by the MSSI general and MSSI neurological subscores (MSSI general, r_τ_ = 0.32; 95%CI, 0.15 to 0.48; p = 2.4 * 10^−4^; MSSI neurological, r_τ_ = 0.32; 95%CI, 0.15 to 0.46; p = 2.7 * 10^−4^). Being employed was negatively related to subjective cognitive complaints (r_τ_ = −0.29; 95%CI, −0.46 to −0.11; p = 4.6 * 10^−3^). MCS and PCS scores were also negatively related to subjective cognitive complaints (MCS, r_τ_ = −0.29; 95%CI, −0.45 to −0.10; p = 6.9 * 10^−4^; PCS, r_τ_ = −0.32; 95%CI, −0.46 to −0.18; p = 1.9 * 10^−4^).

In a proportional odds model, the CESD score (one point increase: OR, 1.07; 95%CI, 1.02 to 1.13; p = 3.3 * 10^−3^), a history of depression (OR, 2.7; 95%CI, 1.1 to 7.3; p = 3.9 * 10^−2^) and the MSSI general score (one point increase: OR, 1.3; 95%CI, 1.1 to 1.5; p = 5.5 * 10^−3^) were independently positively associated with subjective cognitive complaints.

## Discussion

In this large sample of Dutch patients with FD we have, for the first time, shown a relationship between sex, phenotype and risk for OCI. OCI was present in 41% of men with classical disease, affecting mostly the executive functioning domain. In addition, OCI was found in a significant number (27%) of men with non-classical FD. In women with classical FD, however, the prevalence of OCI was markedly lower (7%) and none of the women with non-classical FD had OCI. The risk of OCI in patients with FD was independently related to male sex, a history of stroke and to premorbid IQ.

In a healthy population of male veterans from the United States (n = 4371), slightly younger compared to our cohort (38.4 ± 2.5 years), OCI was found in ~6–7% of this cohort when using similar criteria^8^. In a second healthy mixed control group (n = 138) of a study on the cognitive effects of type 1 diabetes, with comparable age (49 ± 7 years) to our study population, the prevalence of OCI was 5%^9^. This indicates that OCI in our population of FD patients is much higher in male patients than would be expected in this age group. In women with FD the prevalence of OCI is comparable to that in the general population. Loeb *et al*.^7^ found OCI in 30% of patients with FD, but used different criteria to define OCI and a smaller reference population (n = 80), possibly explaining the differences. The impaired executive domain found in our study is in accordance with previous studies in FD^3^. Moreover, our study confirms the preliminary finding of a study by Sigmundsdottir *et al*.^5^ that men with FD are more likely to get OCI, especially in those with classical disease.

In addition, a relationship between the extent of the WMLs (Fazekas score) and the presence of severe OCI was established. In a previous study a subgroup of patients with markedly increased volumes of white matter lesions showed more cognitive deficits compared to patients with lower lesion volumes^6^. In the general population, as was found in our study, the positive relationship between WMLs and OCI is not very strong^10^. It has been postulated that a threshold of WML severity needs to be surpassed before OCI develops^10^.

The relation between stroke and OCI in the general population has been more firmly established^11^. Likewise, in our study the relation between a history of stroke and OCI in FD is clearly present. This was also observed in previous studies in FD, albeit using univariate analyses^5,7^. The positive relationship between (premorbid) IQ and neuropsychological test scores has been firmly established in the general population^8,12^. It has been theorized that a higher premorbid IQ reflects a greater “cognitive reserve”, thus more decline has to take place before OCI occurs^13,14^. The observation that higher T-scores in FD patients with higher premorbid IQ lower the chance of OCI fits this hypothesis. We also found a lower median premorbid IQ in men compared to women. Despite this difference, male sex was related to a higher risk of OCI independently of premorbid IQ. Premorbid IQ therefore does not fully explain the differences in prevalence of OCI between men and women with FD.

A new finding is that almost two-thirds of our cohort of FD patients experienced subjective cognitive complaints, without significant differences in prevalence between all subgroups. Interestingly, in our study the subjective cognitive complaints were not related to OCI, but showed a clear relation with both depression in the past and current depressive symptoms. In the general population, the relation between OCI and subjective cognitive complaints is still controversial^15^. More thoroughly established is that patients with depression have a higher prevalence of subjective cognitive complaints^16^, as was also previously shown by Loeb *et al*.^7^ in a population of FD patients. The relation of depressive symptoms to subjective cognitive complaints further emphasizes the importance of recognizing these symptoms. Conversely, Loeb *et al*.^7^ concluded that in patients with FD prevalence of subjective cognitive complaints is not increased. It seems that, in our cohort of FD patients, subjective cognitive complaints were highly prevalent. Of these, subjective memory complaints were present in 46% of our cohort (data not shown), while these are found in 22% of the general population^17^, indicating that the prevalence in patients with FD could be more than twice as high. The difference to our study might be caused by the difference in assessment (structured interview versus questionnaire) as well as the use of a high cutoff (mean +2 SD compared to a healthy population) for detecting subjective cognitive complaints in the Loeb *et al*.^7^ study. The high prevalence (38%) of depressive symptoms in our study is in line with the previously found prevalence of 46% from a mixed cohort of 186 patients with FD^18^. In populations with chronic diseases or chronic pain various treatments have been shown to improve depressive complaints^19,20^. Unfortunately, treatment effects and risk factors for depression in FD are largely unknown. Only one small, uncontrolled study (n = 15) looked at the effect of psychological counseling and found all FD patients improving^21^. Therefore, efficacy of treatment options for depressive symptoms should be topic of further research in FD patients.

This study has some strengths and limitations. Strengths are the precise phenotyping of the studied cohort and the use of a reliable cognitive test battery. Moreover, this study is the first to combine data on subjective cognitive complaints, depressive symptoms, cognitive functioning and MRI brain parameters in subgroups of patients divided by sex and FD phenotype.

We included 81 patients, more than half of the known patients in the Netherlands, a fairly large group for a rare disease like FD. However, we cannot rule out inclusion bias: patients with subjective cognitive complaints might be more interested in participation. Conversely, patients with severe cognitive impairment might not participate due to participation begin to strenuous. Nevertheless, we found no significant differences in patient characteristics, nor in the presence of TIA, stroke or the height of the Fazekas score between participants and non-participants in this study.

MRIs were made using the same standardized protocol, as part of routine follow up. This means that sometimes there was a time gap between the MRI and the neuropsychological assessment, but in most patients this was not more than a year and WMLs are known to increase slowly. Of some patients MRI of the brain was not available, mostly due to the presence of non-MRI compatible ICD/pacemakers. Excluding these patients, however, might also lead to a bias towards less affected patients.

We did not assess a healthy control group ourselves. Instead, we used large normative datasets (median sample size: 471) compiled of healthy control groups from multiple studies. Furthermore, most neuropsychological test results were corrected for age, sex and education level, although not for (premorbid) IQ.

Lastly, we used the Fazekas-scale to assess WMLs in this study. It has been shown that visual rating scales have a lower ceiling compared to volumetric measurements of WMLs^22^. Perhaps, the use of volumetric measurement of WMLs would have strengthened the relation to OCI.

In conclusion, OCI is present in one-sixth of FD patients, predominantly in men with classical disease. The relation between a history of stroke and OCI in this study re-emphasizes the importance of prevention of stroke in patients with FD. Moreover, the presence of stroke or other clinical indications of OCI warrants referral of FD patients for neuropsychological assessment. The high prevalence of subjective cognitive complaints, equally distributed over the phenotypes and sexes, was not explained by OCI, but showed a clear relation with current or historical depressive complaints. Evaluation of subjective cognitive complaints in patients with FD should therefore include a psychological evaluation and healthcare professionals should focus on recognition and treatment of depressive symptoms.

## Methods

### Study design and phenotype

The baseline data of an ongoing prospective cohort study on neuropsychological functioning in adult patients (≥18 years) with a definite diagnosis of FD^23^ are presented. All known adult Fabry patients (n = 154) at the Academic Medical Centre (AMC), the national referral centre for patients with FD in the Netherlands, were screened for eligibility.

All patients were phenotypically characterized as having classical or non-classical FD^2^, see Supplemental File 1 for criteria. Demographic parameters, clinical and disease characteristics of all patients were gathered from the local Fabry database containing prospectively collected data as well as from medical records.

This study was approved by the Human Research Ethics Committee of the AMC and conducted in accordance with the Declaration of Helsinki in 2013^24^. All participants provided informed consent prior to inclusion and all experiments were performed in accordance with relevant guidelines and regulations. The datasets generated and analyzed during the current study are not publicly available. Because of the rarity of the disease, even anonymized can be linked to a specific individual. In case of a specific scientific question, requests to make part of the dataset available will be reviewed.

### Neuropsychological assessment

All included patients completed a neuropsychological test battery assessing language skills, memory, visuospatial perception, processing speed and executive functioning. Language skills were assessed using the Boston Naming Test (BNT)^25^ and the Wechsler Adult Intelligence Scale IV: Similarities (WAIS-IV: S)^26^. Memory was assessed using the Dutch version of The Rey Auditory Verbal Learning Test (RAVLT)^27^ and the Rivermead Behavioural Memory Test (RBMT): Storytelling^28^, both assessing immediate recall (ir) and delayed recall (dr). Visuospatial perception was assessed using the WAIS IV: Block Design (BD) and the Judgement of Line Orientation (JLO)^26,29^. Processing speed was assessed using the Trail Making Test part A (TMTA)^30^, Stroop Word (W) and Stroop Colour (C)^31^. Executive functioning was assessed using the TMT part B (TMTB)^30^, Stroop Colour-Word (CW)^31^, semantic fluencies, referred to as Animal Fluency and Occupational Fluency^32^ and phonetic fluency, referred to as Letter Fluency^33^.

T-scores (mean of 50, standard deviation (SD) of 10) were calculated per test using extensive Dutch normative data, except for the JLO, for which we used normative data from the United States. The median normative data sample size was 471 healthy participants (range: 121–1000). All normative data were corrected for age and most subtests also for sex and educational level. See Supplemental Table E-1 for additional information on the neuropsychological test battery.

General cognitive functioning was screened using the Mini Mental State Exam (MMSE)^34^. Motivation and underachievement were assessed using the Test of Memory Malingering (TOMM)^35^. An estimation of intelligence was done using the Dutch Adult Reading Test (DART), the Dutch version of the National Adult Reading which correlates strongly with intellectual ability and is relatively resistant to change by neurological impairment^36^.

The neuropsychological test battery was preceded by a structured interview assessing subjective cognitive complaints, addressing the following domains: memory, attention and executive complaints. This resulted in a score ranging from 0 to 3: no subjective cognitive complaints (0), subjective cognitive complaints in one domain (1), in two domains (2) or in all three domains (3), see Supplemental File 2.

### Objective cognitive impairment

OCI was defined as a T-score ≤33 (<5^th^ percentile, −1.67 SD) on at least two neuropsychological tests, resembling statistical significance of two one-tailed tests with p < 0.05. Severe OCI was defined as a T-score ≤30 (<2.3^rd^ percentile, −2 SD) on at least two neuropsychological tests, resembling statistical significance of two two-tailed tests with p < 0.05. We choose the cutoff of −1.67 SD to prevent high rates of false positives, a strategy which has been recommended for other diseases as well^37^. To decrease the family-wise error rate, one or more T-scores ≤33 or ≤30 in the following combination of tests assessing a similar cognitive domain, were regarded as a single abnormal T-score: Animal Fluency and/or Occupational Fluency and/or Letter Fluency, RAVLT ir and/or RBMT ir, RAVLT dr and/or RBMT dr, TMTA and/or Stroop W and/or Stroop C, TMTB and/or Stroop CW, WAIS-IV: BD and/or JLO.

### Questionnaires

*Depressive symptoms* were measured using The Centre for Epidemiological Studies – Depression scale (CESD)^38^. Twenty items on depressive symptoms experienced in the last week are scored on a four point Likert scale (range 0 to 3). Total scores range from 0 to 60 points and patients with scores ≥16 were classified as having depressive symptoms^39^.

*Pain* was measured using the Brief Pain Inventory (BPI) which is divided in three separate sub scores: 1) worst pain, 2) average pain and 3) pain interference with life. The latter is an average score of the influence of pain on general activity, mood, walking, work, enjoyment of life, relations and sleep. Pain scores are graded from 0 (absence of pain) to 10 (worst possible pain)^40^.

*Quality of life* (QoL) was obtained using the Dutch version of the Short Form-36 Health Survey (SF-36). The SF-36 assesses eight domains of QoL which are calculated using Dutch normative data^41^. The eight domains can be grouped into two summary scores: the Physical Component Summary (PCS) and the Mental Component Summary (MCS) that are standardized using normative data from the US population and are presented as T-scores^42^.

*Sleep quality* was measured using The Pittsburgh Sleep Quality Index (PSQI)^43^. The PSQI assesses seven domains which are graded from 0 to 3, resulting in a score ranging from 0 to 21. PSQI scores >5 are indicative for poor sleep quality.

*Fabry Disease severity* was assessed using the Mainz Severity Score index (MSSI, range: 0–76)^44^. The MSSI is composed of four subscales that cover general (range: 0–18), neurological (range: 0–20), renal (range: 0–18) and cardiac (range: 0–20) signs and symptoms of the disease.

### Brain MRI

MRI of the brain was performed yearly or biannually as part of routine follow-up on a 3-T system (Philips Ingenia, Philips Medical Systems, Best, The Netherlands). All MRIs of the brain were made using the same standardized protocol, see Supplemental Table E-2 more information on MRI settings per sequence. Eight patients had no MRI of the brain, six because of the presence of an MRI non-compatible pacemaker or ICD and one because of claustrophobia. In one patient the brain MRI was made in a different hospital. MRIs were re-evaluated by two neuroradiologists, (MRL evaluated basilar artery pathology, MGL evaluated infarctions, WMLs and atrophy), blinded for all patient characteristics. White matter lesions (WMLs) were rated on axial FLAIR using the Fazekas scale, ranging from 0 (no WMLs) to 6 (confluent periventricular and deep WMLs)^45^. Presence and number of (lacunar) infarctions was rated on DWI, axial T2 and FLAIR images. Basilar artery diameter (BAD) was rated on axial T2 images^46^. Atrophy of medial and temporal lobe were rated on T1 3D GRE images using the Medial Temporal lobe Atrophy rating scale (MTA)^47^. See Supplemental Table E-3 for additional information on the scales.

### Statistical methods

Data are presented as median and range or mean ± SD where appropriate. R (version 3.3.1) and SPSS version 24.0 (SPSS Inc. Chicago, Illinois, USA) were used for statistical analysis. Between subgroup differences were compared using the Kruskall-Wallis test, one way ANOVA and Fisher’s exact test where appropriate. Post hoc analyses were done with the Dunn test, Tukeys test and 2 × 2 Fisher’s exact tests with Bonferroni-Holm correction for abovementioned tests, respectively. Included and excluded patients were compared as a whole group and per subgroup divided for sex and phenotype. To check if neuropsychological test results differed from the average from the reference cohort, T-scores were compared to a T-score of 50 using a one-sided sign test.

T-scores per cognitive domain were obtained calculating mean T-scores of all tests addressing this domain. Variables were included in the univariate analyses if they were deemed as potentially related to OCI trough literature search in the general population or previous studies on OCI and FD. Next, the univariate models were used to identify variables for multivariate models. Linear regression was used to analyze the univariate relation between variables and T-scores per cognitive domain. Kendall’s tau-b (r_τ_) was used to analyze the univariate relation between variables and subjective cognitive complaints (range 0–3) and between variables and OCI (binary, yes/no) (Table 6). Bootstrapping was used to calculate bias corrected accelerated 95% confidence intervals (CI) of Kendall’s tau-b, stratified for sex. To minimize the effect of multiple testing, the relation between a variable and OCI was first tested. If the relation between a variable and OCI was (very) weak (≈r_τ_ < 0.1, ≈p > 0.25) a relation between the variable and T-scores per cognitive domain was not tested. To correct for false discovery rate we used the Benjamini-Hochberg procedure in Table 6^48^.

A multiple logistic regression model was used to check which variables are independent risk factors of OCI and a proportional odds model was used to check which variables are independent risk factors of subjective cognitive complaints. Both models were iteratively built selecting variables from univariate models (inclusion if p < 0.10). The Akaike Information Criterion (AIC) was used to optimize the models. We chose the simplest models and compared them to our theoretical concepts. Variance inflation factor was used to explore potential multicollinearity in the logistic regression model. A likelihood ratio test was used to test proportionality of odds, comparing the goodness of fit of the proportional odds model to its multinomial counterpart.

P-values < 0.05 were considered statistically significant. If multiple tests were carried out regarding a single hypothesis, the results were corrected using the Bonferroni-Holm correction, to control for the family wiser error rate^49^.

Results were reported in accordance with the STROBE guidelines^50^.

## Supplementary information

Supplementary files
