# Supplementary material for: Predictors of objective cognitive impairment and subjective cognitive complaints in patients with Fabry disease
Source: Sci Rep. 2019 Jan 17;9:188. doi: 10.1038/s41598-018-37320-0 (PMC6336934; doi:10.1038/s41598-018-37320-0)
Supplement: Supplementary file 1 — Supplementary files [file 41598_2018_37320_MOESM1_ESM.pdf]

Predictors of objective cognitive impairment and subjective cognitive complaints in patients with Fabry disease

Simon Körver[1], MD; Gert J. Geurtsen[2], PhD; Carla E.M. Hollak[1], MD, PhD; Ivo N. van Schaik[3], MD, PhD; Maria G.F. Longo[4], MD, PhD; Marjana R. Lima[5], MD, PhD; Leonardo Vedolin[6], MD, PhD; Marcel G.W. Dijkgraaf[7], PhD; Mirjam Langeveld[1], MD, PhD

[1] *Department of Endocrinology and Metabolism, Academic Medical Center, Amsterdam, The Netherlands*

[2] *Department of Medical Psychology, Academic Medical Center, Amsterdam, The Netherlands*

[3] *Department of Neurology, Academic Medical Center, Amsterdam, The Netherlands*

[4] *Department of Radiology, Massachusetts General Hospital, Boston, The United States*

[5] *Department of Radiology, Hospital Moinhos de Vento, Porto Alegre, Brazil*

[6] *Imaging Section, DASA, São Paulo, Brazil*

[7] *Clinical Research Unit / Department of Clinical Epidemiology, Biostatistics and Bioinformatics, Academic Medical Center, Amsterdam, The Netherlands*

Supplemental file 1

Criteria classical and non-classical Fabry disease

In men, classical disease was defined as: 1) a mutation in the GLA-gene, 2) enzyme activity ≤5% of the mean reference range and 3) ≥1 characteristic FD symptoms (i.e. angiokeratoma, Fabry neuropathic pain, and/or cornea verticillata, see[1] for definitions), or an affected family member with a definite diagnosis according to abovementioned criteria. In women, classical disease was defined as: 1) a mutation in the GLA-gene and 2) ≥1 characteristic FD symptoms, or an affected family member with a definite classical diagnosis according to abovementioned criteria. Men and women, with a mutation in the GLA-gene not regarded as a neutral variant[2], and not fulfilling the criteria for a classical phenotype were diagnosed as having non-classical disease.

References

1. van der Tol L, Cassiman D, Houge G, et al. Uncertain diagnosis of fabry disease in patients with neuropathic pain, angiokeratoma or cornea verticillata: consensus on the approach to diagnosis and follow-up. JIMD Rep 2014;17:83-90.

2. Arends M, Wanner C, Hughes D, et al. Characterization of Classical and Nonclassical Fabry Disease: A Multicenter Study. J Am Soc Nephrol 2017;28:1631-1641.

Supplemental file 2

Structured interview subjective cognitive complaints

SK and GG developed the structured interview in accordance with the neuropsychological history taking, recommended before all neuropsychological test assessments[1]. All structured interviews were conducted by SK or by a neuropsychologist that assisted with data collection.

Methodology

The interview focused on education, work, specific complaints concerning Fabry disease (FD), general medical history, medication, depressive complaints and subjective cognitive complaints.
First, to prevent framing of patients' perception of their own cognition, they were asked broadly about their perceived cognitive functioning. Thereafter all patients were asked the following question:

"Do you have any complaints in the process of thinking (e.g. memory, attention)."

It was verified if these complaints were considered severe to the patient compared to surrounding friends/family/coworkers without Fabry disease. If both questions were answered "Yes" then we considered subjective cognitive complaints as being present. Similar strategies were applied for the different cognitive domains and examples of complaints in specific situations were asked. When doubts were present whether these examples were in agreement with the domain at hand these were explored in more detail.

Reference

1 Strauss, E., Sherman, E. M. S. & Spreen, O. in *A Compendium of Neuropsychological Tests: Administration, Norms, and Commentary* Vol. 3rd Ch. 3, 55-74 (Oxford Univeristy Press, 2006).

Supplemental Table E-1

Table E-1 Neuropsychological test battery

Cognitive domain	Test administered	Scoring
**Intelligence estimation**	Dutch Adult Reading Test[a]	Words correctly read out loud
**Language**	Boston Naming Test	Total correctly recognized drawings
	WAIS-IV: Similarities[b]	Total correct similarities
**Memory**	Rey Auditory Immediate recall[c]	Total immediately recalled words trials 1-5
	Rey Auditory Delayed recall[c]	Total words recalled after 20 minutes
	Rivermead Behavioural Memory Test: Story[c]	Parts of story immediately recalled
	Rivermead Behavioural Memory Test: Story[c]	Parts of story recalled after 15 minutes
**Visuospatial perception**	WAIS-IV: Block Design[b]	Correctly/timely matched patterns
	Judgement of Line Orientation[a]	Correctly matched line pairs
**Processing speed**	Trail Making Test-A[c]	Time to complete
	Stroop Words[c]	Time to complete
	Stroop Colour[c]	Time to complete
**Attention and executive functioning**	Trail Making Test-B[c]	Time to complete
	Stroop Colour-Word[c]	Time to complete
	Fluency Animals[c]	Total numbers of animals in 1 minute
	Fluency Occupation[c]	Total number of occupations in 1 minute
	Fluency Letters[c]	Total number of words with 3 letters 1 minute each

WAIS-IV = Wechsler Adult Intelligence Scale IV

[a] Corrected for age and sex, [b] Corrected for age, [c] Corrected for age, sex and level of education

Supplemental Table E-2

Table E-2 Acquisition parameters for brain MRI

**Parameter**	**T1W 3D GRE**	**FLAIR 3D TSE**	**T2W 2D TSE**	**DWI EPI**
Plane	Sagittal	Sagittal	Axial	Axial
FOV read (mm)	256	250	230	200
FOV phase (%)	100	100	80	100
Slice thickness (mm)	0.9	1.1	3.0	3.0
TR/TE/TI (msec)	9.0/4.1/-	4800/356/1650	4391/80/-	5770/80/-
Flip angle (degree)	8	40	90	90
Bandwidth (Hx/pz)	2.516/172.6	0.594/731.0	1.988/218.5	23.498/18.5

FLAIR = Fluid Attenuated Inversion Recovery; GRE = Gradient Echo; TSE = Turbo Spin Echo; SWI = Susceptibility Weighted Imaging; DWI = Diffusion-Weighted; EPI = Echo Planar Imaging; MOTSA = Multiple overlapping thin slab acquisition; FOV = Field of View; TR = Repetition Time; TE = Echo Time; TI = Inversion Time

Supplemental Table E-3

Table E-3 MRI brain assessment

Pathology	Description	Sequence	Response
WMLs	Presence of WMLs	FLAIR Axial	Yes/no
WMLs	Fazekas<br>periventricular	FLAIR Axial	Fazekas 0: Absence<br>Fazekas 1: "caps" or pencil-thin lining<br>Fazekas 2: Smooth "halo"<br>Fazekas 3: Irregular periventricular hyperintensities extending into deep white matter
WMLs	Fazekas<br>deep white matter	FLAIR Axial	Fazekas 0: None or a single punctate WMH lesion<br>Fazekas 1: Multiple punctate lesions<br>Fazekas 2: Beginning confluency of lesions (bridging)<br>Fazekas 3: Large confluent lesions
Infarctions	Presence of (lacunar) infarctions	T2/FLAIR	Yes/no
Infarctions	Number of (lacunar) infarctions	T2/FLAIR	Number of (lacunar) infarctions
Dilatation basilar artery	Basilar artery diameter (mm)	T2 Axial	Average of:<br>1. Caudal (shortly after the confluence of the vertebral arteries)<br>2. Intermediate (in the middle of the basilar artery)<br>3. Rostral (just before the bifurcation)
Hippocampal atrophy	Medial temporal lobe atrophy rating scale	T1 coronal	0: no CSF is visible around the hippocampus<br>1: choroid fissure is slightly widened<br>2: moderate widening of the choroid fissure, mild enlargement of the temporal horn and mild loss of hippocampal height<br>3: marked widening of the choroid fissure, moderate enlargement of the temporal horn, and moderate loss of hippocampal height<br>4: marked widening of the choroid fissure, marked enlargement of the temporal horn, and the hippocampus is markedly atrophied and internal structure is loss

WMLs = White matter lesions, FLAIR = Fluid Attenuated Inversion Recovery

Supplemental Table E-4

Table E-4 Characteristics non-participants

	All	Men		Women	
		Classical	Non-classical	Classical	Non-classical
Patients, n (%)	73	18 (24.7%)	5 (6.8%)	34 (46.6%)	16 (21.9%)
Age in years, mean (±SD)	47.5 (±17.9)	38.3 (±14.4)	58.8 (±20.7)	50.0 (±17.8)	48.8 (±18.1)
History of cerebral event#, n (%)	9 (12.3%)	2 (11.1%)	3 (60.0%)	4 (11.8%)	0 (0.0%)
History of TIA#, n (%)	6 (8.2%)	2 (11.1%)	1 (20.0%)	3 (8.8%)	0 (0.0%)
History of stroke#, n (%)	6 (8.2%)	1 (5.6%)	2 (40.0%)	3 (8.8%)	0 (0.0%)
Fazekas					
Total score (0-6), median (range)	1 (0-6)	1 (0-6)	0.5 (0-1)	1 (0-6)	0 (0-1)

Continuous variables are presented as median (range) or mean (±SD), discrete variables as number (percentages).

TIA = Transient Ischemic Attack, #As diagnosed by a neurologist
